# Alternative splicing regulation by tumor suppressing subtransferable candidate 4: a pathway to tumor suppression

**DOI:** 10.3389/fimmu.2024.1474527

**Published:** 2024-12-04

**Authors:** Haiping Zhao, Nana Wu, Gaigai Wei, Huiling Zhang, Tingrong Ren, Jingjing Yi, Yuqi Zhang, Zixi Wang, Yihan Wang, Zhihan Guo, Duanwu Zhang

**Affiliations:** Children’s Hospital of Fudan University, National Children’s Medical Center, and Shanghai Key Laboratory of Medical Epigenetics, International Colaboratory of Medical Epigenetics and Metabolism, Ministry of Science and Technology, Institutes of Biomedical Sciences, Fudan University, Shanghai, China

**Keywords:** TSSC4, alternative splicing, splicing, gene expression, tumorigenesis, cancer progression, oncogene

## Abstract

**Introduction:**

RNA splicing is a crucial posttranscriptional process that governs gene expression, and defects in alternative splicing contribute to various diseases, including cancer. Tumor suppressing subtransferable candidate 4 (TSSC4) is a known tumor suppressor and has been identified as part of the U5 small nuclear ribonucleoprotein (snRNP), which is involved in tri-snRNP biogenesis. However, the precise role of TSSC4 in regulating alternative splicing and its impact on tumor growth remain unclear.

**Methods:**

To explore the link between splicing modulation and tumor suppression driven by TSSC4, we conducted transcriptome sequencing (RNA-seq) on TSSC4-knockout and wild-type HeLa cells. Additionally, we analyzed alternative splicing and gene expression in various cancer cell lines, including TSSC4-knockout A549 cells and TSSC4-knockdown PANC-1, MDA-MB-231, and MCF-7 cells. Splicing patterns and gene expression profiles were compared between TSSC4-deficient and control cells.

**Results:**

Our RNA-seq analysis revealed that TSSC4 deficiency in HeLa cells results in widespread alterations in splicing patterns and gene expression. Specifically, the loss of TSSC4 led to abnormal alternative splicing events and dysregulation of tumor-associated genes, including several oncogenes. This effect was confirmed across multiple cancer cell lines, highlighting a consistent role of TSSC4 in splicing regulation.

**Discussion:**

These findings demonstrate that TSSC4 plays a crucial role in regulating RNA splicing, particularly in controlling the splicing of many oncogenes. Our results reveal a novel mechanism by which TSSC4 mediates tumor suppression through the modulation of alternative splicing, which could provide implications for understanding TSSC4’s role in cancer biology.

## 1 Introduction

Post-transcriptional regulation is a crucial aspect of gene expression control at the mRNA level and encompasses the regulation of mRNA stability, translation, modification, localization, and alternative splicing (AS) (1). Alternative splicing allows a single pre-mRNA to be spliced into diverse mature transcripts, resulting in transcriptomic and proteomic diversity. As one of the most extensive mechanisms of gene regulation, alternative splicing occurs in the vast majority of human genes and precisely controls gene expression at the co- and post-transcriptional levels across various tissues and developmental stages. Aberrant regulation of alternative splicing is closely associated with numerous human diseases, including cancer (2, 3).

Alternative splicing can be categorized into five major events: skipped exons (SE), retained introns (RI), alternative 5’ splice sites (A5SS), alternative 3’ splice sites (A3SS), and mutually exclusive exons (MXE) (4). RNA splicing is a highly regulated process that requires the spliceosome, along with cis-acting elements and trans-acting proteins. The spliceosome is a large multimeric ribonucleoprotein (RNP) complex whose composition and structure dynamically change during pre-mRNA splicing (5). Small nuclear RNAs (snRNAs), together with associated proteins, form small nuclear ribonucleoprotein particles (snRNPs), which are the fundamental building blocks of the spliceosome. The spliceosome consists of five main small nuclear ribonucleoproteins (snRNPs): U1, U2, U4, U5, and U6 snRNPs (6, 7). The U4/U6. U5 tri-snRNP is the largest preassembled spliceosomal complex (8). Four of the U5-specific proteins—PRPF8, SNRNP200, EFTUD2, and SNRNP40—form a stable RNA-free heterotetrameric complex (RHC) (9).

TSSC4 (tumor suppressing subtransferable candidate 4) is a component of the U5 small nuclear ribonucleoprotein (snRNP) and associates with the U5 snRNP and the PRPF19 complex through two distinct conserved domains (10, 11). As a mono-U5 snRNP-specific protein, TSSC4 binds to the subunits of the RNA-free heterotetrameric complex (RHC) during their biogenesis in the cytoplasm and remains associated until the complete U5 snRNP is formed (11). In the absence of TSSC4, U5 snRNP accumulates in Cajal bodies, but this U5 particle is unable to interact with the U4/U6 di-snRNP to form the functional tri-snRNP. This deficiency leads to the accumulation of U4, U5, and U6 snRNAs in this nuclear structure. Therefore, TSSC4 plays a crucial role in promoting the formation of tri-snRNPs, ensuring proper spliceosome assembly and function.

Furthermore, TSSC4 directly interacts with the SNRNP200 (U5 snRNP 200 kDa helicase) and the PRPF8 spliceosomal scaffold. During U5 snRNP or U4/U6.U5 tri-snRNP assembly and recycling, these interactions may help prevent SNRNP200 from unwinding or translocating non-cognate RNAs or avert premature SNRNP200-mediated U4/U6 di-snRNA unwinding (12).

TSSC4 is associated with several human diseases, including Beckwith–Wiedemann syndrome (BWS), Wilms’ tumor 2 (WT2), and other embryonal cancers, as well as lung, breast, and ovarian cancer (13). It has been reported that TSSC4 inhibits cancer cell and tumor growth and prevents cell death during excessive growth by inhibiting autophagy (14). TSSC4 knockdown has been shown to increase cell proliferation in the breast cancer cell line MDA-MB-231, the glioblastoma multiforme (GBM) cell lines U87 and U373, and the cervical cancer cell line HeLa (14). However, the molecular and cellular mechanisms of TSSC4 in cancer have not been clearly elucidated.

In this study, we aimed to investigate how TSSC4 affects cell proliferation and tumor progression. Our studies revealed that TSSC4 plays a critical role in the regulation of alternative splicing of many tumor-associated genes. These findings uncover a previously unrecognized function of TSSC4 in cancer cell progression, highlighting its potential as a promising target for therapeutic intervention through the modulation of alternative splicing in cancer treatment.

## 2 Results

### 2.1 Loss of TSSC4 causes transcriptome-wide alterations in gene expression

**Figure 1:**
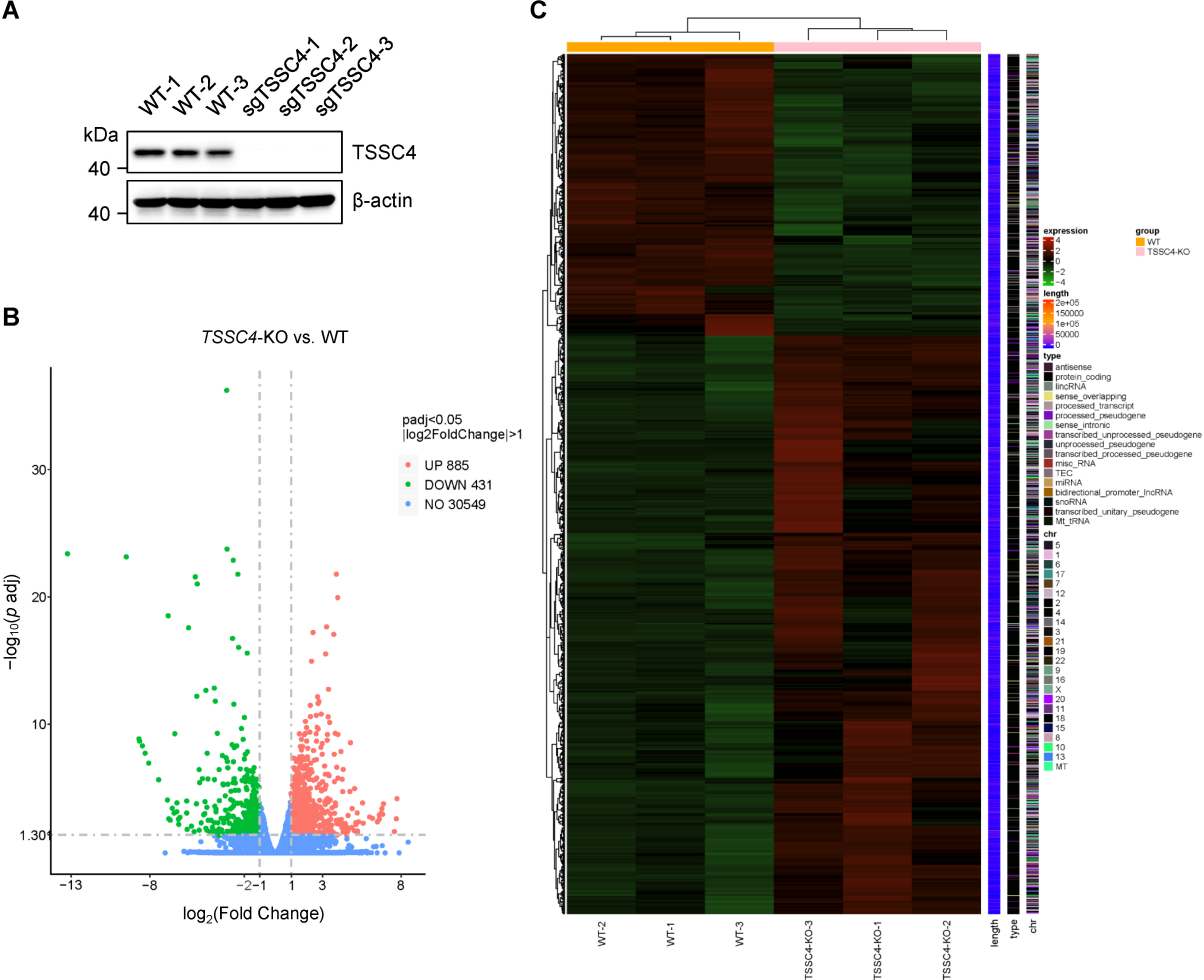
Loss of tumor suppressing subtransferable candidate 4 causes transcriptome-wide changes in gene expression. **(A)** HeLa cells were infected with a lentivirus (lentiCRISPR-v2) encoding *TSSC4* sgRNA (sg*TSSC4*). After puromycin selection, single cell lines were selected for validation of *TSSC4* knockout (*TSSC4*-KO). Immunoblotting was used to assess TSSC4 protein expression in three *TSSC4*-KO HeLa single clone cells and wild-type HeLa cells used for RNA-seq. β-Actin was used as an internal control. The uncropped source images for the Western blots are presented in **Supplementary Presentation 1**. **(B)** Volcano plots indicating the relative expression levels of genes whose expression was downregulated and upregulated in *TSSC4*-KO cells compared with that in wild-type HeLa cells, as revealed by RNA-seq. **(C)** Heatmap of differentially expressed genes (DEGs) between *TSSC4*-KO and wild-type HeLa cells revealed by RNA-seq. TSSC4, tumor suppressing subtransferable candidate 4.

Given that TSSC4 is a component of the U5 snRNP and that its deficiency results in significant alterations in tri-snRNP formation, it is likely that cells experience aberrant kinetics of spliceosome assembly. Considering the interdependent relationship between splicing and transcription, such spliceosome insufficiency may lead to widespread changes in gene expression and pre-mRNA splicing patterns. To investigate this, we performed RNA sequencing (RNA-seq) using *TSSC4*-knockout (KO) HeLa cells (deposited in the GEO repository, accession number GSE273634). Transcriptome profiling revealed that 1,316 genes presented abnormal expression levels, with 885 genes upregulated and 431 genes downregulated in *TSSC4*-KO cells compared with control cells (**Figures 1A, B**, **Supplementary Data Sheet 1**). The differentially expressed genes (DEGs) between the *TSSC4*-KO and control groups were analyzed via a heatmap to visualize gene expression patterns (**Figure 1C**).

**Figure 2:**
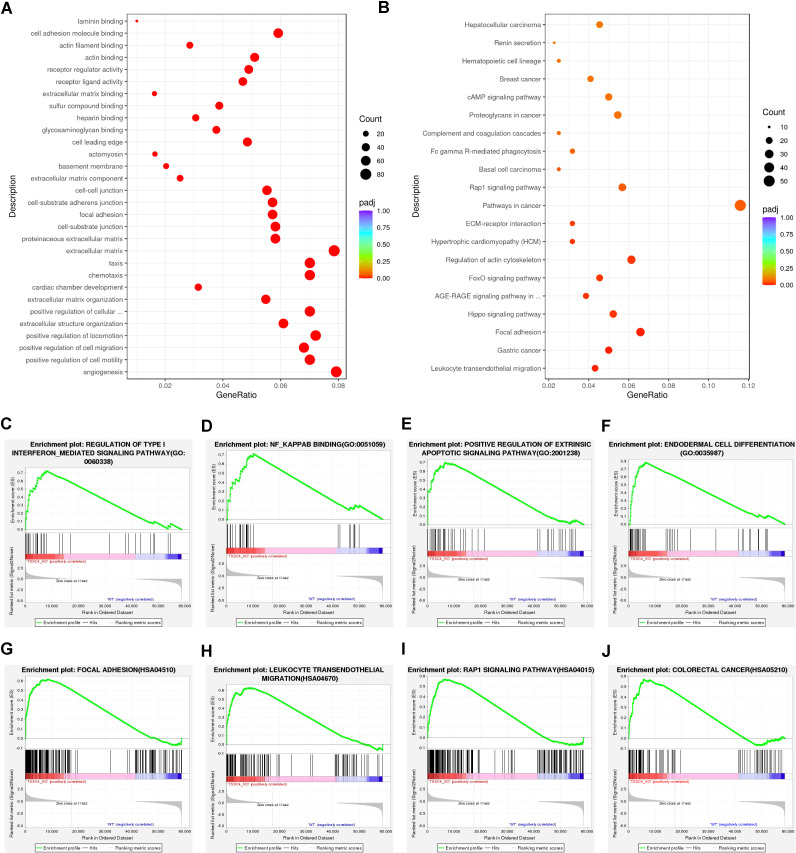
Functional enrichment analysis of differentially expressed genes in tumor suppressing subtransferable candidate 4 knockout vs. wild-type HeLa cells. **(A)** Gene Ontology (GO) analysis of genes differentially expressed between *TSSC4*-KO and wild-type HeLa cells, as revealed by RNA-seq. **(B)** Kyoto Encyclopedia of Genes and Genomes (KEGG) enrichment analysis of genes differentially expressed between *TSSC4*-KO and wild-type HeLa cells, as revealed by RNA-seq. **(C−F)** Gene Set Enrichment Analysis (GSEA) for the GO pathways. The GSEA results showed that the terms ‘regulation of type I interferon-mediated signaling pathway’, ‘NF-κB binding’, ‘positive regulation of extrinsic apoptotic signaling pathway’, and ‘endodermal cell differentiation’ were enriched in the *TSSC4*-KO phenotype. **(G−J)** Representative GSEA results for the KEGG pathways. The GSEA results showed that the terms ‘focal adhesion’, ‘leukocyte transendothelial migration’, ‘Rap1 signaling pathway’ and ‘colorectal cancer’ were enriched in the *TSSC4*-KO phenotype. TSSC4, tumor suppressing subtransferable candidate 4.

Functional annotation through Gene Ontology (GO) enrichment analysis revealed that DEGs in TSSC4-deficient cells were significantly associated with angiogenesis, cell motility, migration, chemotaxis, the extracellular matrix, and focal adhesion (**Figure 2A**). Furthermore, Kyoto Encyclopedia of Genes and Genomes (KEGG) pathway enrichment analysis revealed the associations of the DEGs with focal adhesion, pathways in cancer, leukocyte transendothelial migration, and the MAPK signaling pathway (**Figure 2B**). To further explore TSSC4-related signaling pathways in cancer cells, we performed Gene Set Enrichment Analysis (GSEA) for the GO pathways and KEGG pathways and identified signaling pathways related to the regulation of type-I interferon, NF-κB binding, focal adhesion, colorectal cancer, the Rap1 signaling pathway, and leukocyte transendothelial migration. These pathways are important for cancer-related functions in the *TSSC4*-KO group (**Figures 2C−J**).

### 2.2 TSSC4 deficiency leads to significant abnormalities in alternative splicing

**Figure 3:**
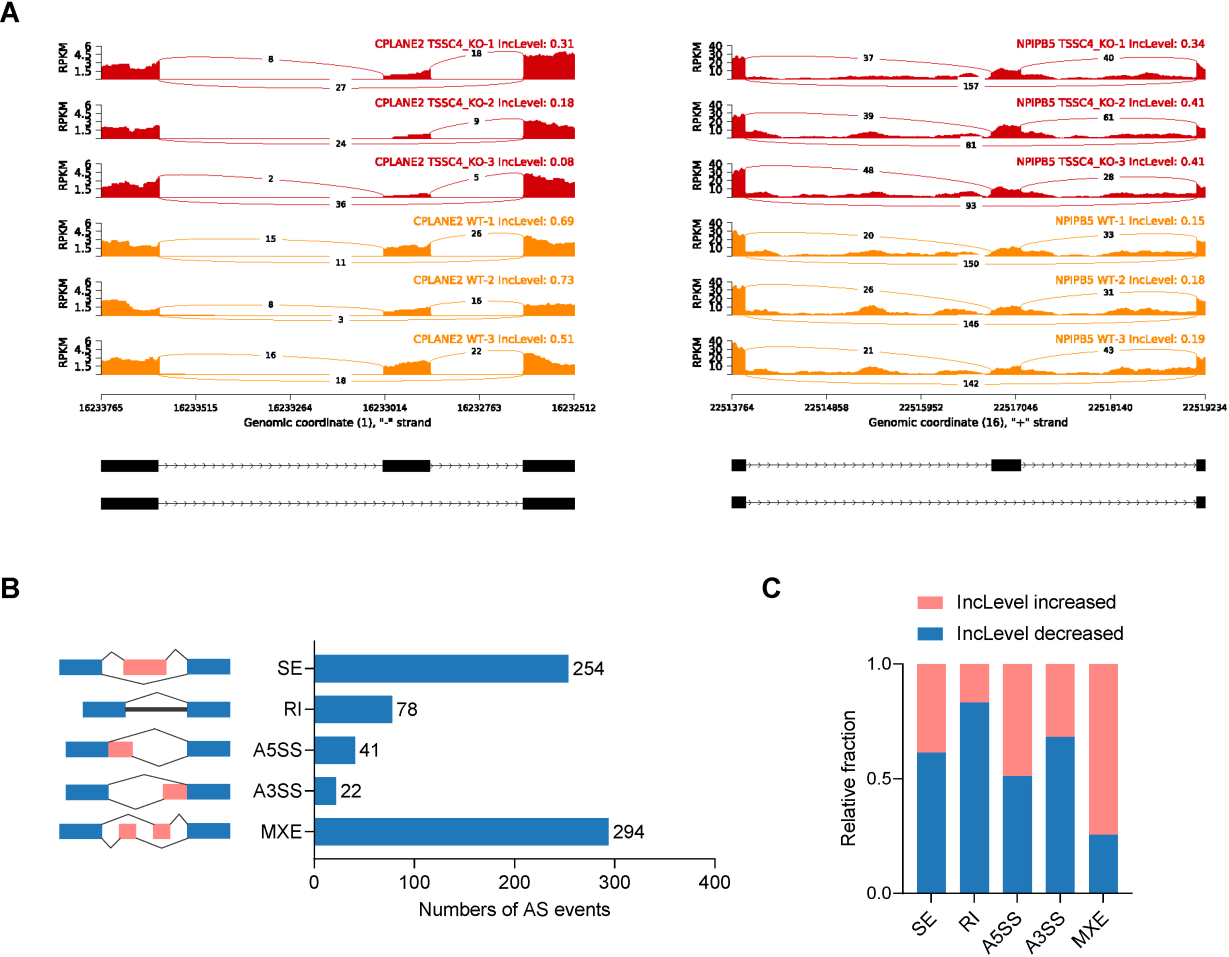
Tumor suppressing subtransferable candidate 4 deficiency results in aberrant gene splicing. **(A)** Examples of alternative exons affected by TSSC4 deficiency in HeLa cells revealed by RNA-seq. Genes were chosen to represent both an increase and a decrease in the IncLevel (Inclusion Level), and the numbers of exon junction reads are indicated. **(B)** Quantification of the different alternative splicing events induced by TSSC4 depletion. The left panel represents the five types of alternative splicing, and the right panel indicates their respective quantities. **(C)** Relative fraction of each alternative splicing event affected positively or negatively by TSSC4 deficiency. TSSC4, tumor suppressing subtransferable candidate 4.

Significant changes in gene expression are accompanied by changes in splicing patterns. To gain insights into the impact of TSSC4 on alternative splicing (AS) events, we conducted an alternative splicing analysis. Read tracks for two representative examples of AS events are shown in **Figure 3A**. Overall, we identified 689 AS events across all known types (**Figure 3B**). Subsequent analysis revealed that TSSC4 deficiency resulted in significant aberrant alternative splicing events, which are either positively or negatively regulated by TSSC4 (**Figure 3C**).

**Figure 4:**
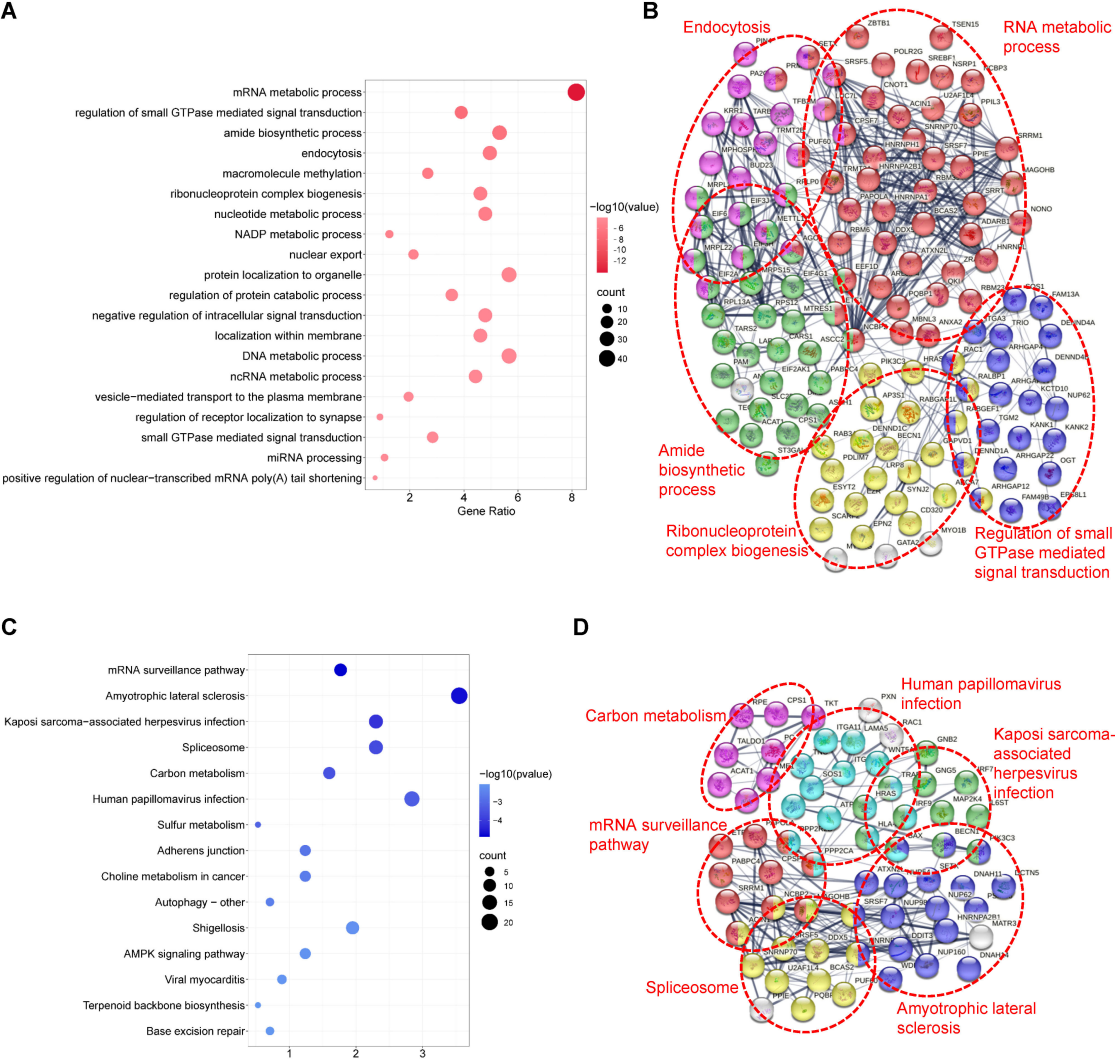
Functional enrichment analysis of differential alternative splicing events in tumor suppressing subtransferable candidate 4 knockout vs. wild-type HeLa cells. **(A)** Gene Ontology (GO) analysis of TSSC4-regulated alternative splicing events in HeLa cells revealed by RNA-seq. **(B)** Functional association network of TSSC4-regulated alternative splicing events. The genes in **(A)** were analyzed using the Search Tool for the Retrieval of Interacting Genes/Proteins (STRING) database. **(C)** Kyoto Encyclopedia of Genes and Genomes (KEGG) enrichment analysis of TSSC4-regulated alternative splicing events in HeLa cells revealed by RNA-seq. **(D)** Functional association network of TSSC4-regulated alternative splicing events. The genes in **(C)** were analyzed using the STRING database. TSSC4, tumor suppressing subtransferable candidate 4.

GO analysis revealed that TSSC4-regulated AS changes were notably enriched in RNA metabolic processes, small GTPase-mediated signal transduction, and ribonucleoprotein complex biogenesis (**Figure 4A**). Additionally, an assessment using the Search Tool for the Retrieval of Interacting Genes/Proteins (STRING) indicated that many of the splicing targets regulated by TSSC4 were functionally connected to a well-linked interaction network (**Figure 4B**).

**Figure 5:**
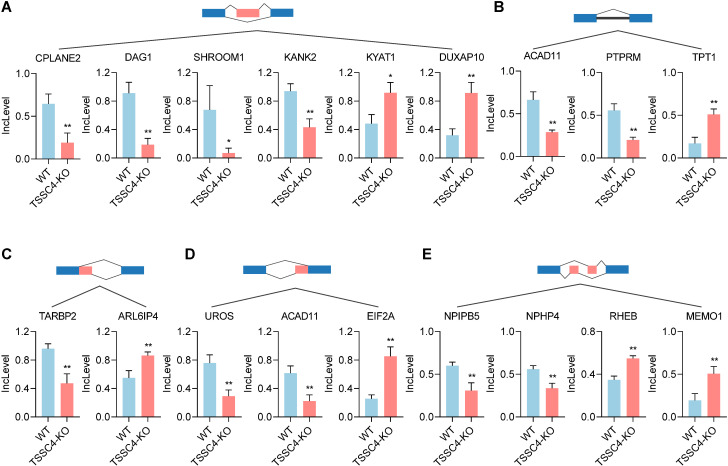
Changes in various alternative splicing events caused by tumor suppressing subtransferable candidate 4 depletion. **(A−E)** Representative examples of different types of alternative splicing events in *TSSC4*-KO vs. wild-type HeLa cells revealed by RNA-seq. (**(A)** SE; **(B)** RI; **(C)** A5SSI; **(D)** A3SS; **(E)** MXE). *P* values were calculated using an unpaired Student’s *t* test. Data are presented as mean ± SD. *P < 0.05, **P < 0.01, ***P < 0.001. SE, skipped exons; RI, retained introns; A5SS, alternative 5’ splice sites; A3SS, alternative 3’ splice sites; MXE, mutually exclusive exons. TSSC4, tumor suppressing subtransferable candidate 4.

KEGG pathway analysis of TSSC4-regulated AS changes revealed that TSSC4 influences splicing events in several key pathways, including the mRNA surveillance pathway, spliceosome, amyotrophic lateral sclerosis, and Kaposi sarcoma-associated herpesvirus infection (**Figure 4C**). Further interaction networks of KEGG pathways were determined via STRING (**Figure 4D**). Several examples of alternative splicing events were identified from the RNA sequencing results (**Figures 5A−E**). For skipped exons (SE), the deletion of *TSSC4* resulted in a significant reduction in the IncLevel (Inclusion Level) of several genes, specifically CPLANE2 (decreased by 0.455), DAG1 (decreased by 0.724), SHROOM1 (decreased by 0.608), and KANK2 (decreased by 0.509). Conversely, there was an increase in the IncLevels of KYAT1 (increased by 0.433) and DUXAP10 (increased by 0.595) (**Figure 5A**). For retained introns (RI), *TSSC4* deletion led to decreased IncLevels for ACAD11 (decreased by 0.381) and PTPRM (decreased by 0.348), whereas TPT1 exhibited an increase in IncLevel (increased by 0.340) (**Figure 5B**). With respect to alternative 5’ splice sites (A5SS), *TSSC4* deletion resulted in a decrease in TARBP2’s IncLevel (decreased by 0.489) alongside an increase in ARL6IP4’s IncLevel (increased by 0.314) (**Figure 5C**). With respect to alternative 3’ splice sites (A3SS), *TSSC4* deletion caused a decrease in the IncLevels of UROS (decreased by 0.465) and ACAD11 (decreased by 0.394), whereas EIF2A showed a significant increase in IncLevel (increased by 0.598) (**Figure 5D**). Finally, in the analysis of mutually exclusive exons (MXE), *TSSC4* loss resulted in a reduction in the IncLevels for NPIPB5 (decreased by 0.292) and NPHP4 (decreased by 0.222). Conversely, RHEB (increased by 0.199) and MEMO1 (increased by 0.312) demonstrated increased IncLevels following *TSSC4* deletion (**Figure 5E**). Taken together, these results indicate that TSSC4 dysfunction leads to splicing abnormalities, which is consistent with the role of TSSC4 in modulating tri-snRNP formation.

### 2.3 TSSC4 deficiency impacts the alternative splicing and expression of tumor-associated genes

**Figure 6:**
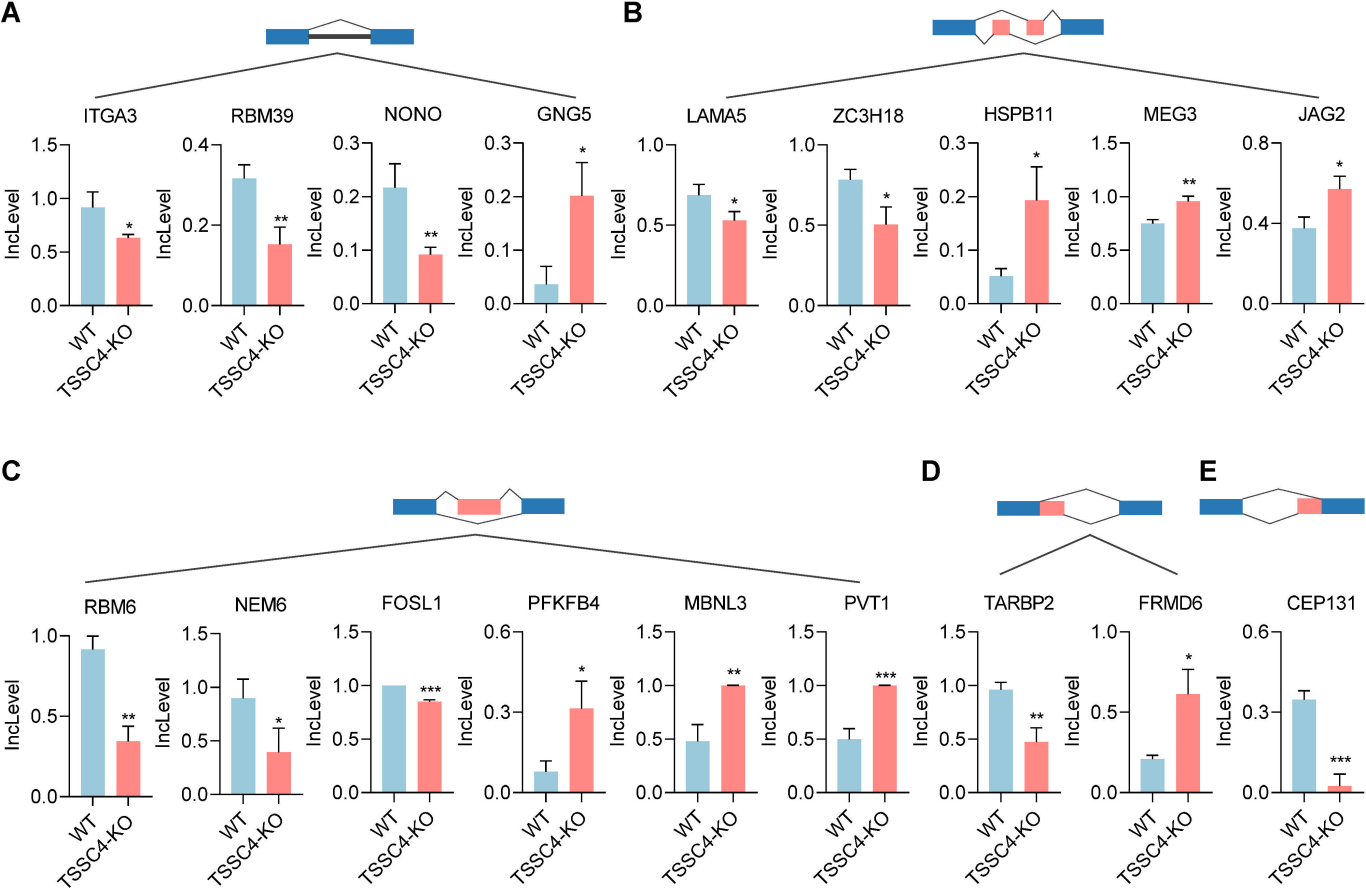
Loss of tumor suppressing subtransferable candidate 4 induces alternative splicing abnormalities in tumor-associated genes. **(A−E)** Representative different types of alternative splicing events in tumor-associated genes in *TSSC4*-KO vs. wild-type HeLa cells revealed by RNA-seq. (**(A)** RI; **(B)** MXE; **(C)** SE; **(D)** A5SS; **(E)** A3SS). *P* values were calculated using an unpaired Student’s *t* test. Data are presented as mean ± SD. *P < 0.05, **P < 0.01, ***P < 0.001. RI, retained introns; MXE, mutually exclusive exons; SE, skipped exons; A5SS, alternative 5’ splice sites; A3SS, alternative 3’ splice sites. TSSC4, tumor suppressing subtransferable candidate 4.

Previous studies have shown that TSSC4 functions as a tumor suppressor, inhibiting cancer cell and tumor growth. It is well recognized that the upregulation of many oncogenic proteins accelerates cancer cell proliferation. To elucidate the role of TSSC4 in tumor growth, we evaluated its impact on the alternative splicing of genes associated with tumorigenesis (**Figures 6A−E**). For retained introns (RI), the deletion of *TSSC4* resulted in a significant decrease in the IncLevels of several tumor-regulatory genes, such as ITGA3 (decreased by 0.284), RBM39 (decreased by 0.164) and NONO (decreased by 0.125). Conversely, GNG5 exhibited an increase in IncLevel (increased by 0.166) (**Figure 6A**). For mutually exclusive exons (MXE), *TSSC4* deletion led to reduced IncLevels for LAMA5 (decreased by 0.161) and ZC3H18 (decreased by 0.279), while concurrently increasing the IncLevel of HSPB11 (increased by 0.142), MEG3 (increased by 0.208) and JAG2 (increased by 0.197) (**Figure 6B**). With respect to skipped exons (SE), *TSSC4* deletion resulted in decreased IncLevels for RBM6 (decreased by 0.572), NME6 (decreased by 0.502), and FOSL1 (decreased by 0.202). Conversely, there was an increase in the IncLevels of PFKFB4 (increased by 0.236), MBNL3 (increased by 0.519) and PVT1 (increased by 0.502) (**Figure 6C**). For alternative 5’ splice sites (A5SS), *TSSC4* deletion resulted in a decrease in TARBP2’s IncLevel (decreased by 0.489) and an increase in FRMD6’s IncLevel (increased by 0.404) (**Figure 6D**). Additionally, for alternative 3’ splice sites (A3SS), *TSSC4* deletion caused a decrease in the IncLevel of CEP131 (decreased by 0.322) (**Figure 6E**). Our results show that TSSC4 deficiency results in notable alternative splicing abnormalities in many tumor-associated genes that could potentially facilitate cancer progression.

**Figure 7:**
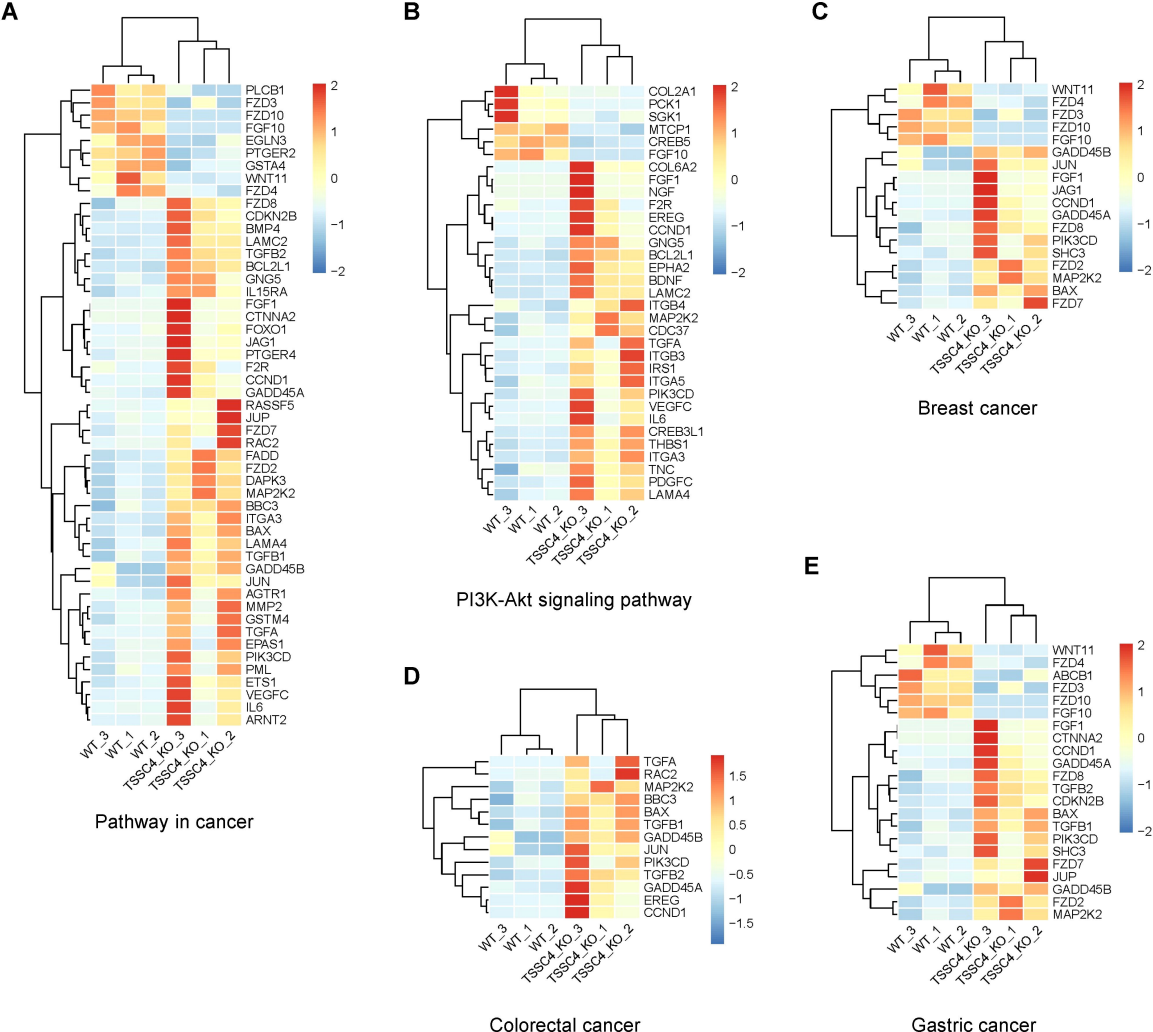
Heatmaps of the altered genes in the tumor-associated Kyoto Encyclopedia of Genes and Genomes pathways. **(A−E)** Heatmaps of the differentially expressed genes between *TSSC4*-KO and wild-type HeLa cells for the tumor-associated KEGG pathways, as revealed by RNA-seq. **(A)** Pathway in cancer; **(B)** PI3K-Akt signaling pathway; **(C)** Breast cancer; **(D)** Colorectal cancer; **(E)** Gastric cancer. Normalized FPKM (Fragments Per Kilobase of transcript per Million mapped reads) values were used to represent gene expression levels, where each column corresponds to a biological replicate of a sample and each row represents a specific gene. The data for each gene have been normalized, with a color scale indicating the fold change. KEGG, Kyoto Encyclopedia of Genes and Genomes.

**Figure 8:**
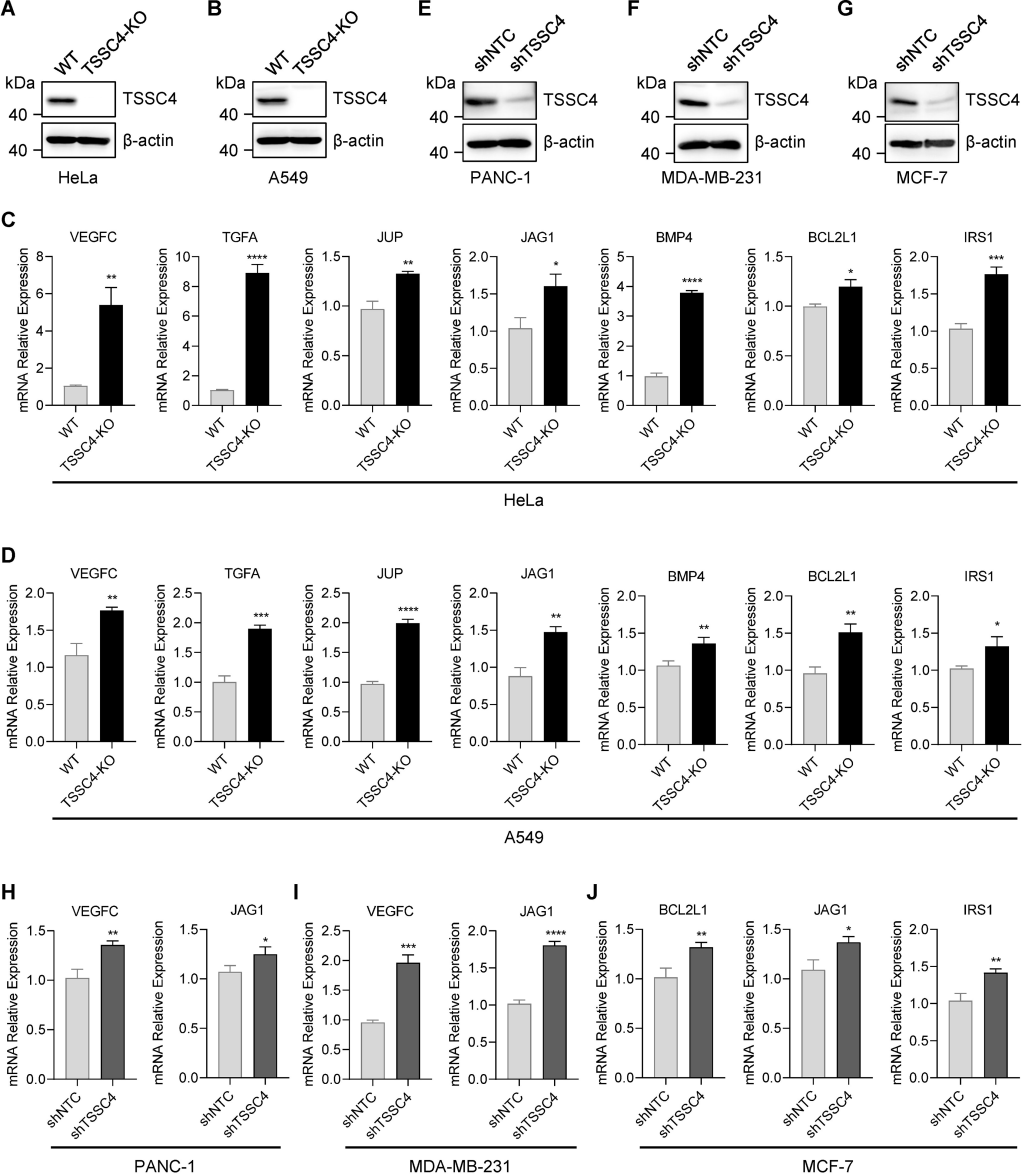
Tumor suppressing subtransferable candidate 4 deficiency leads to increased expression of multiple oncogenes. **(A, B)** HeLa and A549 cells were infected with a lentivirus (lentiCRISPR-v2) encoding *TSSC4* sgRNA (sg*TSSC4*). After puromycin selection, single cell lines were selected for validation of the absence of TSSC4. Immunoblotting showing TSSC4 expression levels in *TSSC4*-KO and wild-type HeLa **(A)** and A549 **(B)** cell lines. β-Actin was used as a loading control. **(C, D)** RT−qPCR analysis of several candidate oncogenes in *TSSC4*-KO and wild-type HeLa cells **(C)** and A549 cells **(D)**. Quantification was calculated relative to β-Actin (ACTB) level. **(E−G)** PANC-1, MDA-MB-231, and MCF-7 cells were infected with a lentivirus encoding *TSSC4* shRNA (sh*TSSC4*) or nontargeting control shRNA (shNTC). Immunoblotting showing TSSC4 expression levels in *TSSC4*-knockdown and control PANC-1 cells **(E)**, MDA-MB-231 cells **(F)**, and MCF-7 cells **(G)** at 72 hours after lentiviral infection. **(H−J)** RT−qPCR analysis of representative oncogenes in *TSSC4*-knockdown and control PANC-1 cells **(H)**, MDA-MB-231 cells **(I)**, and MCF-7 cells **(J)**. *P* values were calculated by unpaired two-tailed Student’s *t* tests. Data are presented as mean ± SD. *P < 0.05, **P < 0.01, ***P < 0.001, ****P < 0.0001 **(C, D, H–J)**. The uncropped source images for the Western blots are presented in **Supplementary Presentation 1**. The source data for the RT−qPCR are presented in **Supplementary Data Sheet 4**. TSSC4, tumor suppressing subtransferable candidate 4.

To further investigate the effects of TSSC4 downregulation on oncogenic proteins that promote cancer cell growth, we visualized the up- and down-regulated gene expression within tumor-associated KEGG pathways via heatmaps (**Figures 7A−E**). We validated the upregulation of several oncogenes in *TSSC4*-KO HeLa and A549 cells (**Figures 8A−D**). Additionally, we confirmed these findings in several *TSSC4*-knockdown cancer cell lines, including a pancreatic cancer cell line (PANC-1) and two breast cancer cell lines (MDA-MB-231 and MCF-7) (**Figures 8E−J**).

Collectively, these results demonstrate that TSSC4 deficiency leads to aberrant alternative splicing and increased expression of oncogenes, further supporting the role of TSSC4 as a tumor suppressor.

## 3 Discussion

The perturbed expression of splicing factors and dysregulated splicing are molecular hallmarks of cancer. Alternative splicing plays a critical role in driving tumorigenesis (15). In our study, we investigated the role of TSSC4, a component of the U5 small nuclear ribonucleoprotein (snRNP), which promotes tri-snRNP formation and acts as a novel tumor suppressor by inhibiting cell proliferation and tumor growth (11, 14). Through RNA sequencing of TSSC4 knockout and control HeLa cells, we elucidated the molecular mechanism underlying the inhibitory role of TSSC4. Gene ontology analysis revealed that the differentially expressed genes were significantly associated with angiogenesis, cell migration, and the extracellular matrix, supporting the role of TSSC4 in tumor development. We confirmed that TSSC4 knockout led to increased expression of several oncogenes, such as VEGFC (16), JAG1 (17), BCL2L1 (18) and IRS1 (19), which have been confirmed to promote tumor progression by enhancing cell proliferation or inhibiting apoptosis. These findings suggest that TSSC4 influences the expression of many cancer-associated genes through the regulation of alternative splicing.

Our findings indicate that TSSC4 regulates global alternative splicing events, with *TSSC4* knockout increasing the number of retained introns, mutually exclusive exons and skipped exons, highlighting its crucial role in maintaining alternative splicing fidelity. In certain contexts, mRNAs with retained introns are subject to rapid degradation via the nonsense-mediated decay (NMD) pathway, which effectively decreases gene expression and allows cells to finely regulate protein production levels. In our study, the deletion of TSSC4 resulted in a reduction in intron retention for ITGA3 (Integrin alpha 3), leading to a corresponding increase in its protein expression. ITGA3, a member of the integrin family, is frequently characterized as an oncogenic factor across various tumor types. It facilitates cancer progression by enhancing cell migration and invasion, modulating the tumor microenvironment, and activating tumor-associated signaling pathways, including the PI3K/AKT and MAPK pathways. As a result, ITGA3 is recognized as a potential therapeutic target in cancer treatment, warranting further investigation into its role in tumor biology and therapeutic interventions (20). Exon skipping also serves as a critical mechanism for regulating gene expression by altering the stability, localization, and translation efficiency of mRNA transcripts. Some skipped-exon variants are less stable or subject to degradation, thereby providing a means of down-regulating protein levels. In our study, *TSSC4* knockout resulted in an increase in exon skipping of RBM6 (RNA binding motif protein 6), which may lead to a decrease in RBM6 protein expression. RBM6 is an RNA-binding protein involved in various cellular processes, including RNA splicing, transcriptional regulation, and modulation of gene expression. Research suggests that RBM6 exerts a tumor-suppressive role across multiple cancer types (21). It is involved in the regulation of key cancer-related signaling pathways, such as the p53 and PI3K/AKT pathways, which are critical for controlling cell proliferation, apoptosis and invasion. The loss or mutation of RBM6 can lead to aberrant splicing of certain tumor-associated genes, thereby facilitating tumorigenesis and progression. Additionally, in both HeLa and A549 cells, *TSSC4* knockout led to increased expression of several oncogenes. These findings suggest that TSSC4 influences the expression of many cancer-associated genes through the regulation of alternative splicing.

Despite these insights, there are limitations to our study that warrant discussion. First, the focus on certain types of cancer cell lines may not fully capture the diverse roles of TSSC4 in other cancer types. Future studies should investigate TSSC4’s function across a broader range of cell lines and primary cancer samples to validate its role in different tumor contexts. Additionally, while our transcriptomic sequencing identified genes with significant expression changes, further research is needed to determine the precise mechanisms by which TSSC4 regulates specific alternative splicing events. The potential for context-dependent regulation by TSSC4 means that the findings in these few cell lines may not be universally applicable.

Moreover, while our study revealed a correlation between TSSC4 and splicing regulation, it did not conclusively demonstrate a direct interaction between TSSC4 and pre-mRNA. Future experiments could employ techniques such as RNA immunoprecipitation and CRISPR-based approaches to elucidate the direct targets and mechanisms by which TSSC4 influences splicing. In addition to splicing regulation, TSSC4 has also been shown to suppress cancer cell growth by inhibiting autophagy (14). Depletion of TSSC4 in EGFRvIII-expressing glioblastoma multiforme (GBM) cells shifted the function of autophagy from a pro-cell survival role to a pro-cell death role during prolonged cell growth. Autophagy is a conserved intracellular degradation process that functions to recycle redundant proteins and malfunctioning organelles (22). It has opposite and context-dependent functions in cancer cell growth, depending on various biological environments (23). Autophagy promotes tumor progression by providing nutrients to cells to support cancer cell growth (24), but when over-activated by severe stresses, it can induce cell death (25). Future investigations should be conducted to determine whether the splicing regulation by TSSC4 contributes to its ability to suppress autophagy and how this process interacts with splicing regulation.

In conclusion, our study provides evidence for the important role of TSSC4 in mediating cancer progression through the regulation of alternative splicing and the expression of tumor-associated genes. Further investigation is necessary to clarify the molecular mechanisms involved. Additional studies in specific cancer types may explore whether modulating alternative splicing by targeting TSSC4 could be broadly applied as a novel therapeutic strategy for cancer.

## 4 Materials and methods

### 4.1 Cell culture

HeLa, PANC-1, MDA-MB-231, MCF-7, and HEK293T cell lines were maintained in Dulbecco’s modified Eagle’s medium (DMEM) supplemented with 10% fetal bovine serum, 100 IU/ml penicillin and 100 μg/ml streptomycin and cultured in a humidified incubator at 37°C with 5% CO_2_. A549 cells were maintained in Ham’s F-12K medium supplemented with 10% fetal bovine serum and penicillin/streptomycin. There were no signs of mycoplasma contamination in any of the cell lines. Cell line authentication was performed via short tandem repeat profiling.

### 4.2 Plasmid constructs

To knock out *TSSC4* in HeLa and A549 cells, a small guide RNA (sgRNA) targeting the TSSC4 gene (5’-caaagatgtcacggctgcgc-3’) was inserted into the lentiCRISPR-v2 vector (Addgene, Plasmid # 52961). To knock down *TSSC4* in various cancer cell lines, including PANC-1, MDA-MB-231, and MCF-7 cells, short hairpin RNA (shRNA) targeting *TSSC4* (shTSSC4) (5’-CGGTGGTGCTGAAGTGGAA-3’) and nontargeting control shRNA (shNTC) (5’-CAACAAGATGAAGAGCACC-3’) were cloned into the pLV-H1-EF1a-puro lentiviral vector (Biosettia, Cat. # SORT-B19). All the plasmid constructs were validated by DNA sequencing. The lentiviral vectors were transduced into the cancer cell lines via the lentivirus-mediated system described below.

### 4.3 Lentiviral packaging and infection

Lentivirus was produced with HEK293T cells by transfecting the lentiviral vectors and packaging plasmids (pVSV-G, pMDL g/p RRE, and pRSV-Rev) using a calcium phosphate-mediated transfection system. Stable cell lines were established via lentiviral infection followed by puromycin selection.

### 4.4 RNA sequencing and data analysis

Total RNA was purified from *TSSC4*-KO and wild-type HeLa cells using TRIzol reagent (Invitrogen) according to the manufacturer’s protocol for the subsequenrt library preparation. Poly(A) mRNA isolation was performed using Oligo(dT) beads. The RNA-seq library was loaded on an Illumina NovaSeq 6000 instrument for sequencing using a 2×150 paired-end (PE) configuration according to manufacturer’s instructions. The mapped reads were aligned to the hg38 reference genome by HISAT2 (v2.0.5) for further analysis. Differential expression analysis of the two groups was performed using the edgeR R package (3.22.5). The P values were adjusted using the Benjamini & Hochberg method. To determine significant differential expression, the following thresholds were applied: P adj ≤ 0.05 and |Log2(fold change) | ≥ 1. Alternative splicing events were identified mainly by rMATS (version 4.1.0). ψ = (I/LI)/(I/LI + S/LS), ψ = Inclusion Level (IncLevel), I = number of reads mapped to the exon inclusion isoform (IC_SAMPLE_1), S = number of reads mapped to the exon skipping isoform (SC_SAMPLE_1), LI = effective length of the exon inclusion isoform (IncFormLen), and LS = effective length of the exon skipping isoform (SkipFormLen). The differentially expressed genes and alternative splicing events are listed in **Supplementary Data Sheet 1** and **Supplementary Data Sheet 2**, respectively.

### 4.5 Western blot

Proteins in the cell lysates were quantified using a BCA assay and separated by SDS−polyacrylamide gel electrophoresis. The proteins were then transferred to a nitrocellulose membrane (Cytiva) and blocked with 5% nonfat milk in Tris-buffered saline. Immunoblotting was performed using the indicated antibodies. Next, the membrane was incubated with a horseradish peroxidase (HRP)-linked secondary antibody at room temperature for 1 hour. Detection was carried out using an electrochemiluminescence (ECL) system according to the manufacturer’s instructions. The primary antibodies used for immunoblotting were anti-TSSC4 (Cat. # 14531-1-AP) and anti-β-Actin (Cat. # 60008-1-Ig) from Proteintech.

### 4.6 RNA extraction, reverse transcription, and quantitative real-time PCR (RT−qPCR)

TRIzol reagent (Invitrogen) was used for total RNA extraction according to the manufacturer’s instructions. HiScript III RT SuperMix for qPCR Kit (Vazyme) and 2x SYBR Green qPCR master mix (Vazyme) were used for RNA reverse transcription and RT−qPCR, respectively. ACTB served as an internal control. Fold changes were calculated by relative quantification (2^–ΔΔCt^). The primers used for RT−qPCR are listed in **Supplementary Data Sheet 3**.

### 4.7 Statistical analysis

Prism 8 (GraphPad Software) was used to determine the statistical significance of the data. Comparisons between two groups were made via an unpaired, two-tailed Student’s *t* test. Differences with *P* < 0.05 were considered statistically significant.

## Data Availability

The RNA-seq data presented in this study are deposited in the GEO repository, accession number GSE273634. All the other data supporting the findings of the paper are available from the corresponding author upon reasonable request.
