# Supplementary material for: Alternative splicing regulation by tumor suppressing subtransferable candidate 4: a pathway to tumor suppression
Source: Front Immunol. 2024 Dec 4;15:1474527. doi: 10.3389/fimmu.2024.1474527 (PMC11652373; doi:10.3389/fimmu.2024.1474527)
Supplement: Supplementary file 5 [file Presentation1.pptx]

## Slide 1
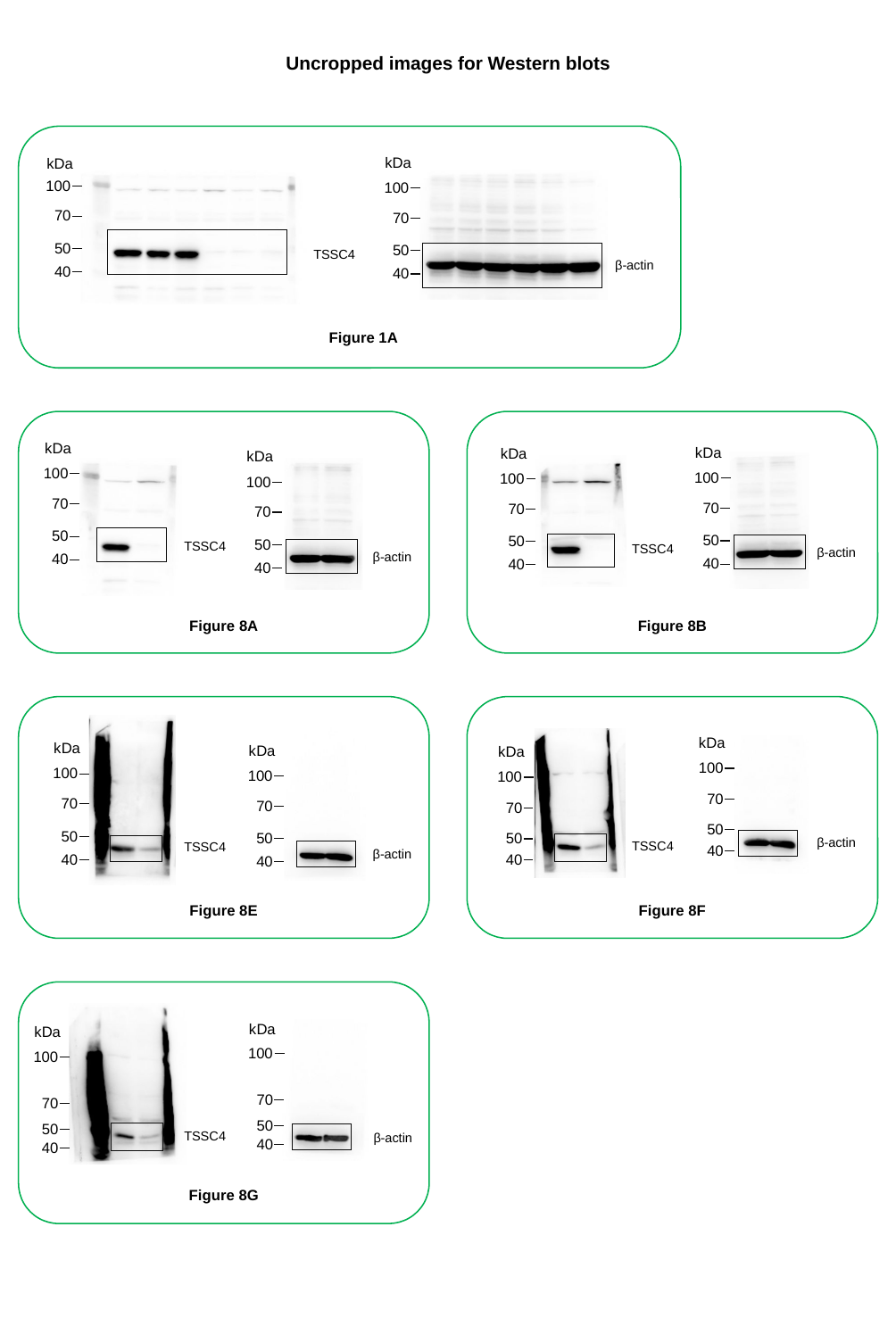

Uncropped images for Western blots
kDa
100
70
50
40
kDa
100
70
50
TSSC4
β-actin
40
Figure 1A
kDa
100
70
50
40
kDa
100
70
50
40
kDa
100
70
50
40
kDa
100
70
50
40
TSSC4
TSSC4
β-actin
β-actin
Figure 8A
Figure 8B
kDa
100
70
50
40
kDa
100
70
50
40
kDa
100
70
50
40
kDa
100
70
50
40
β-actin
TSSC4
TSSC4
β-actin
Figure 8E
Figure 8F
kDa
100
70
50
40
kDa
100
70
50
40
TSSC4
β-actin
Figure 8G
